# Randomised, controlled clinical trial evaluating the effects of preoperative insomnia treatment on postoperative pain control and recovery: a protocol for the Promoting Sleep to Alleviate Pain-Arthroplasty (PROSAP-A) trial

**DOI:** 10.1136/bmjopen-2025-099785

**Published:** 2025-07-30

**Authors:** Martin F Bjurström, Kristoffer Bothelius, Pernilla Maathz, Susanna Jernelöv, Martin Kraepelien, Alexander H C Rosenström, Andrea Niklasson, Michael T Smith, Richard Olmstead, Michael R Irwin, Patrick H Finan, Eva Kosek

**Affiliations:** 1 Clinical Pain Research, Department of Surgical Sciences, Uppsala University, Uppsala, Sweden; 2 Department of Psychiatry and Biobehavioral Sciences, University of California Los Angeles, Los Angeles, California, USA; 3 Centre for Psychiatry Research, Department of Clinical Neuroscience, Karolinska Institutet, Stockholm, Sweden; 4 Department of Psychiatry and Behavioral Sciences, Johns Hopkins University, Baltimore, Maryland, USA; 5 Department of Psychiatry and Biobehavioral Sciences, David Geffen School of Medicine, University of California Los Angeles, Los Angeles, California, USA; 6 Department of Anesthesiology, University of Virginia School of Medicine, Charlottesville, Virginia, USA; 7 Department of Clinical Neuroscience, Karolinska Institute, Stockholm, Sweden

**Keywords:** SLEEP MEDICINE, Pain management, SURGERY, Insomnia

## Abstract

**Introduction:**

Sleep is a biological necessity with vital effects on all tissues and organs of the body. Preoperative sleep disturbance is associated with increased postoperative pain intensity and opioid consumption. Given that insomnia is a potentially modifiable risk factor, interventions targeting sleep prior to surgery may improve postoperative pain control and enhance key outcomes of recovery.

**Methods and analysis:**

Promoting Sleep to Alleviate Pain-Arthroplasty (PROSAP-A) is a randomised, parallel group, two arm, controlled trial evaluating the effects of preoperative sleep-promotion on postoperative pain control, brain health and physical recovery. The main objective is to investigate whether preoperative insomnia treatment in patients scheduled to undergo total knee arthroplasty (TKA) or total hip arthroplasty (THA) may improve acute postoperative pain control. 100 adults with insomnia disorder (Insomnia Severity Index score >10 and confirmed Diagnostic and Statistical Manual of Mental Disorders, Fifth Edition criteria for persistent insomnia disorder), scheduled to undergo primary TKA or THA, will be randomised to preoperative cognitive behavioural therapy for insomnia (CBT-I) or an active comparator control intervention, sleep education therapy (SET). Both interventions will be delivered over 4 weeks in hybrid format through a digital self-guided platform in combination with weekly telehealth video sessions with a psychologist (CBT-I) or research nurse (SET). A video-assisted booster session will be provided 1–2 weeks postoperatively. The primary outcome measure is acute postoperative pain intensity, averaged over the first 7 postoperative days (POD). Secondary outcome measures include long-term postoperative pain control, changes in quantitative sensory testing variables (eg, temporal summation, conditioned pain modulation), sleep, cognition (eg, attention, memory, processing speed, executive function), mental health, health-related function, physical activity, quality of life and blood biomarkers. Participants will undergo on-site evaluation preoperative (preintervention and postintervention) and 6 months postoperative. Additional remote assessments will take place during POD1–7, 3 and 12 months postoperative.

**Ethics and dissemination:**

The Swedish Ethical Review Authority has approved the PROSAP-A trial protocol. Results will be published in international peer-reviewed journals and summaries will be provided to funders and participants of the trial.

**Trial registration number:**

NCT06145516.

**STRENGTHS AND LIMITATIONS OF THIS STUDY**

- Intervention will be delivered prior to orthopaedic surgery, as a prehabilitation approach, for the treatment of insomnia.
- Unmeasured confounding will be mitigated by a randomised, outcome-assessor blinded, active comparator design.
- Intervention efficiency, accessibility and scalability will be maximised by self-guided digital-based delivery methods.
- Potential mechanisms of action will be informed by multimodal assessments of perioperative sleep and domains related to postoperative recovery.
- Study generalisability will be challenged by participant enrolment and adherence in the context of major orthopaedic surgery.

## Introduction

 Sleep and pain are bidirectionally related. Although pain can make it harder to fall asleep and trigger frequent awakenings, the causality is even stronger in the opposite direction: sleep problems predict development, aggravation and spreading of pain.^1^,^3^ Experimental studies examining different models of sleep loss in animals and humans have consistently shown that sleep disturbance lowers pain thresholds, evokes hyperalgesia and sometimes even causes spontaneous pain.^4^,^11^

Given that there is now a solid evidence base supporting the close relationship between sleep and pain, research focus has gradually shifted towards trying to characterise specific mechanisms that may mediate the effects of sleep disturbance on pain perception and pain neurophysiology. For example, in a randomised clinical trial, we have shown that two nights of profound sleep fragmentation, through decreased slow-wave sleep, with subsequent increases in cellular inflammation, mediates a large proportion of the observed heightened heat pain sensitisation.^12^ In a pharmacoexperimental part of the same study, it was shown that sleep fragmentation leads to attenuated morphine analgesia during cold pressor pain testing.^13^ Moreover, Haack *et al* recently demonstrated that extended experimental sleep loss increases monocytic expression of cyclooxygenase 2 in healthy volunteers, with sustained increases even after recovery sleep.^14^

Based on the intimate interaction between sleep and pain, it is not surprising that an increasing number of studies show that preoperative sleep problems are associated with impaired acute postoperative pain control^15^ and development of chronic postsurgical pain (CPSP).^16^ Since different types of sleep disturbances may be targeted as potentially modifiable risk factors, perioperative pharmacological sleep intervention has been trialled, and in some cases shown to decrease acute postoperative pain intensity and lower opioid consumption.^17^ Pharmacological sleep promotion, in particular through melatonin, also shows promising pain-reducing effects in persistent pain populations.^18^

In addition to the negative effects related to pain, it is well known that sleep disturbance, especially loss of deep sleep (slow-wave sleep), negatively impacts the immune system, healing and DNA-repair processes which increase the risk of several chronic diseases, including osteoporosis, dementia, cardiovascular disease and cancer.^19^ Sleep problems are greatly over-represented among patients with persistent pain,^20^ for example, patients with osteoarthritis. Hence, these patients constitute a highly vulnerable group during the perioperative phase of major surgery.

Cognitive behavioural therapy for insomnia (CBT-I) is the first-line treatment for insomnia disorder.^21^ In contrast to pharmacotherapies, there are no risks of tolerance or dependence associated with this psychological treatment and the effect duration extends well beyond the end of the intervention.^22^ In a recent meta-analysis, Selvanathan *et al* found that CBT-I targeted to patients with persistent pain conditions and comorbid insomnia significantly improves sleep, but only weakly decreases pain intensity.^23^ One challenge in clinical trials of CBT-I for chronic pain is variability in when the intervention is delivered relative to pain symptom trajectories. Therefore, a potential advantage of delivering CBT-I in the perioperative period is that all participants will receive the intervention within a similar time frame around a discrete event—surgery—which may reduce heterogeneity and improve the precision of measurement of pain trajectories. Although perioperative interventions can be challenging to implement, meta-analytical data show that other perioperative psychological treatments are feasible and may beneficially impact both acute and long-term postoperative pain outcomes.^24^

Given that recovery after total knee arthroplasty (TKA) and total hip arthroplasty (THA) is commonly painful and burdensome, with relatively high risk for significant lingering pain symptoms and CPSP,^25^,^27^ preoperative identification and targeting of patients with osteoarthritis with severe comorbid sleep problems constitutes a novel treatment strategy. Hence, the main objective of the PROSAP-A (Promoting Sleep to Alleviate Pain-Arthroplasty) trial is to investigate whether preoperative sleep promotion may lead to improved acute postoperative pain control. Based on the global, essential effects of sleep on virtually all tissues and organs of the body, our secondary hypothesis is that preoperative targeting of sleep will improve multiple other outcome measures and domains, including central sensitisation, inflammation, postoperative recovery, cognition, mental health (lower degree of anxiety and depression symptoms), health-related function and quality of life.^19 28^ As these measures may contribute to the estimation of treatment effects, our multimodal outcome assessments at major time points will facilitate innovative exploratory analyses of treatment moderators.

## Methods and analysis

### Study overview

This study protocol is reported according to the SPIRIT (Standard Protocol Items for Randomised Trials) statement.^29^ Briefly, PROSAP-A is a perioperative randomised, controlled trial with a 12-month follow-up period after TKA or THA, aiming to investigate both acute and long-term postoperative effects of preoperative sleep promotion. The main objective is to evaluate whether preoperative insomnia treatment in patients with severe knee or hip osteoarthritis and comorbid insomnia, scheduled to undergo primary TKA or THA, improves acute postoperative pain control. A pilot study evaluating study flow and procedures is currently ongoing (start date October 2024) and will include a total of 10 participants. The randomised, controlled PROSAP-A trial is planned to start in September 2025, with an estimated date of study completion by December 2030.

### Study setting

On-site study visits will take place at the Multidisciplinary Pain Center at Uppsala University Hospital (Uppsala, Sweden). Surgeries will be performed at the Elisabeth Hospital (Uppsala, Sweden), and the hospital in Enköping (Sweden), which are both located in urban settings.

### Eligibility criteria

Patients must provide written, informed consent before any study procedures occur.

#### Inclusion criteria

Patients eligible for the trial must comply with all of the following at randomisation:

- Age ≥18 years.
- Insomnia Severity Index (ISI) score >10.
- Fulfil Diagnostic and Statistical Manual of Mental Disorders, Fifth Edition (DSM-V) criteria for persistent insomnia disorder.
- Average pain Numerical Rating Scale (NRS) score ≥4 (scale 0–10) and/or movement-related pain NRS score ≥4 after 5 min walking.
- Scheduled to undergo primary (first-time, ie, not revision surgery) TKA or THA due to osteoarthritis.

Note that patients who have previously undergone THA or TKA in the contralateral hip or knee joint will be considered for inclusion.

#### Exclusion criteria

- Uncontrolled medical disorders.
- Night shift work.
- Ongoing major depressive disorder, bipolar disorder, psychotic disorder, substance dependence.
- Current history or high likelihood of primary sleep disorders (other than insomnia), including obstructive sleep apnoea syndrome, narcolepsy and nocturnal myoclonus.
- Severely impaired vision (precluding ability to take part in study interventions).

### Anaesthesia, surgery and perioperative care

Although the mode of anaesthesia will be standardised (THA typically spinal anaesthesia through intrathecal injection of hyperbaric bupivacaine, TKA typically through general anaesthesia), anaesthesia protocol data will be collected for each surgery. THA will be performed through a posterior approach. THA and TKA will be conducted as cemented, hybrid or uncemented procedures, according to surgeon preference. All surgeries will be performed in the setting of a modern, fast-track enhanced recovery after surgery programme, including early mobilisation on the day of surgery and multimodal pain control (eg, COX-2 inhibitor in combination with paracetamol and oxycodone prn). Perioperative care at both centres complies with recommendations presented in recent PROSPECT (procedure-specific postoperative pain management) publications.^30 31^

### Interventions

The sleep-promoting interventions were selected based on efficiency, prior research experience and with the potential to be implemented in clinical practice, to optimise the level of adherence in a real-world setting.

#### Cognitive behavioural therapy for insomnia

The CBT-I will focus on the two components, sleep restriction therapy and stimulus control, which have shown the highest efficacy for sleep improvement among components typically incorporated.^32^ The treatment will consist of a 4-week self-guided digital, online (web app) CBT-I intervention (provided by the eHealth core facility at Karolinska Institutet, according to Jernelöv *et al*^33^), coupled with once weekly (four total) telehealth video-consultations with a psychologist (up to a maximum of 60 min per session) to enhance effects and compliance. Recent meta-analytical data show that effects of telehealth administration of CBT-I are comparable with individual on-site CBT-I.^34^ A telehealth CBT-I booster session will be administered 1–2 weeks postoperative.

Over the course of the sleep restriction therapy, participants will use a digital sleep registration tool to set and gradually adjust their ‘sleep window’ to increase sleep efficiency and find an optimal balance between sleep time and sleep efficiency. Sleep restriction therapy may in the short term increase daytime sleepiness, reinforcing the importance of safety guidelines when delivering sleep restriction therapy (eg, avoid driving),^35^ but over the course of a few weeks, sleep restriction therapy is very effective in improving insomnia, and is a—if not the—core component of CBT-I.^36^ The stimulus control component aims to strengthen the association between the bed and sleep, by minimising the time spent awake in bed and maximising the time spent asleep in bed.

#### Sleep education therapy

When evaluating psychological interventions, it is important to include an active comparator control treatment.^24^ In this trial, we will be using sleep education therapy (SET), administered in digital self-guided form over a 4-week period. Topics of the SET include sleep physiology, sleep disorders, general health and ageing, complementary medical approaches, health factors relating to sleep and sleep hygiene measures. This type of control condition has previously been found adequate in CBT-I trials.^28^ In addition to the digital format, telehealth video consultations with a research nurse will be provided once weekly (up to a maximum of 60 min per session) over the 4 weeks. The two research nurses involved in the study will receive adequate pretrial training related to sleep physiology, sleep hygiene and behavioural changes, to enable effective support of study participants. Identical to the CBT-I intervention, a telehealth SET booster session will be administered 1–2 weeks after surgery. Briefly, the educational information will describe learning objectives and patient activities to promote integration of the material. SET is known to produce modest benefits in insomnia, although not as robust or durable as for CBT-I.

An overview of the key components included in the digital interventions is provided in table 1.

**Table 1:** An overview of the key components included in the digital interventions

Components of sleep education therapy (4 weeks)	Components of CBT-I (FastAsleep) (4 weeks)
Digital tool: notebook.	Sleep registration and sleep window adjustment tool: a simplified sleep diary, logs bedtime, rise time, time awake during the night and subjective sleep quality. Sleep efficiency is calculated automatically for every registered night. After every four registered nights, the patient is invited to adjust their sleep window, that is, maximum of six adjustments.
Education component week 1: myths about sleep. Sleep hygiene education. The types of sleep problems.	Module 1: Psychoeducation regarding sleep and sleep difficulties; sleep hygiene information; rationales for stimulus control and sleep restriction therapy. Introduction of patient narratives.
Education component week 2: the biology of sleep. Stress and the immune system, in relation to sleep.	Module 2: Short module focusing only on common problems and suggestions for solutions, encouragement to carry on despite possibly increased daytime sleepiness. Follow example patients on their journey towards better sleep.
Education component week 3: medical conditions that can interfere with your sleep. Dreams and nightmares.	Module 3: Short module focusing only on continued problem solving and encouragement to carry on even if improvement has yet to occur. Follow example patients on their journey towards better sleep.
Education component week 4: psychological treatments for sleep problems. Alcohol, benzodiazepines and other sleep aids.	Module 4: Short module focusing only on continued problem solving and encouragement to carry on even if improvement has yet to occur. Follow example patients on their journey towards better sleep.
(No maintenance plan)	Module 5: Maintenance plan and advice on management of possible setbacks and relapses. Follow example patients on their journey towards better sleep.

CBT-I, cognitive behavioural therapy for insomnia.

Significant efforts will be made to retain study participants in the assigned intervention group; measures include text notifications, e-mails and phone calls, as needed. Moreover, the support provided by psychologists/research nurses is designed to maximise retention.

### Outcomes

**Figure 1:**
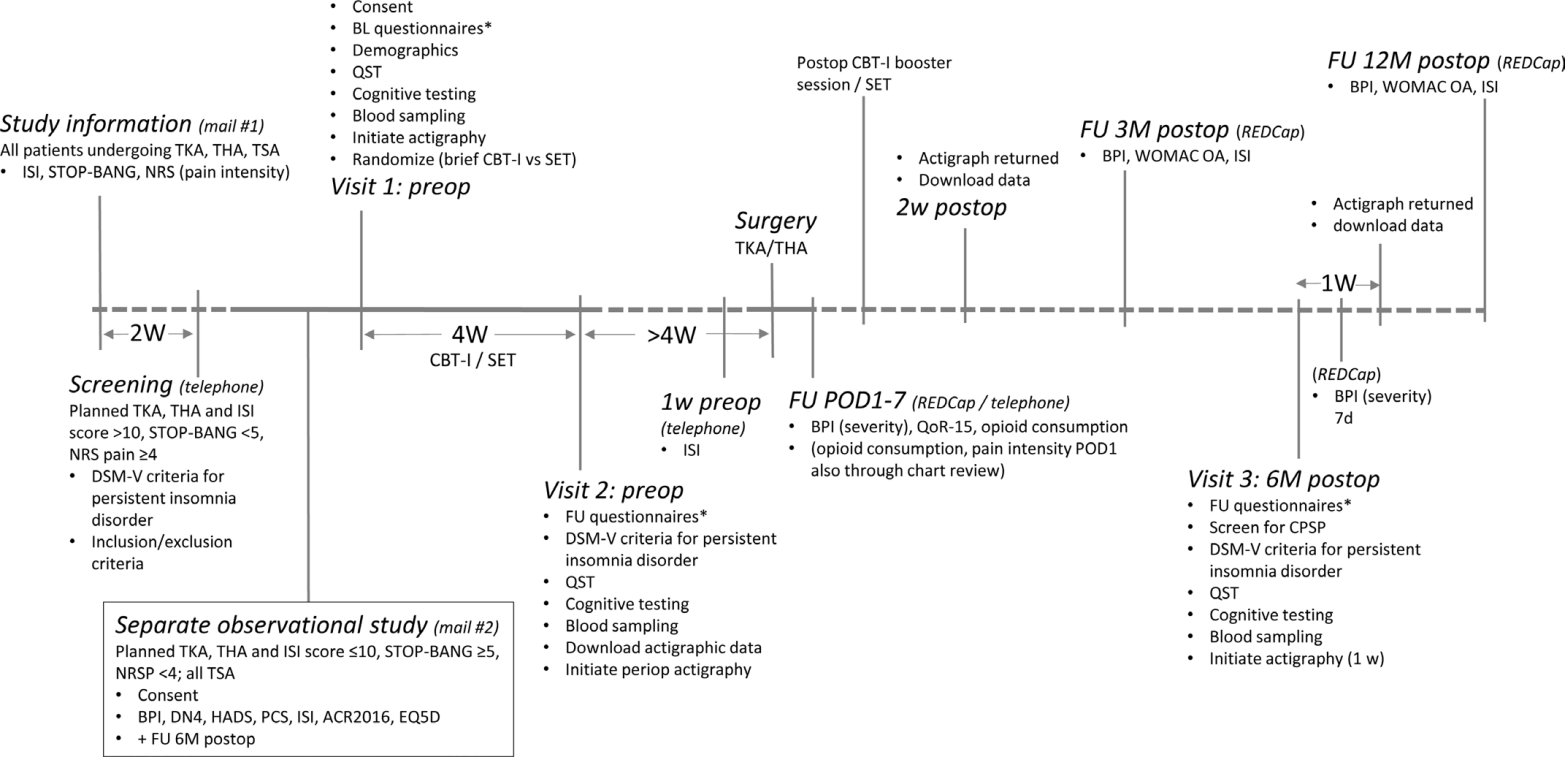
Timing of outcome assessment. *Questionnaires include: ACR2016, BPI-sf, CPAQ-8, DN4, EQ-5D, ESS, GAD-7, IPAQ-sf, ISI, PCS, PHQ-9, PSQ, PSQI, RAND-36, TSK, WOMAC OA. ACR2016, American College of Rheumatology 2016 fibromyalgia diagnostic criteria; BL, baseline; BPI, brief pain inventory; CBT-I, cognitive behavioural therapy for insomnia; CPAQ-8, 8-item Chronic Pain Acceptance Questionnaire; CPSP, chronic postsurgical pain; d, days; DN4, Douleur Neuropathique 4; DSM, Diagnostic and Statistical Manual of Mental Disorders; EQ-5D, EuroQol 5 dimensions questionnaire; FU, follow-up; GAD-7, Generalised Anxiety Disorder scale 7; HADS, Hospital Anxiety and Depression Scale; IPAQ-sf, International Physical Activity Questionnaire short form; ISI, Insomnia Severity Index; M, months; NRS, Numerical Rating Scale; PCS, Pain Catastrophising Scale; periop, perioperative; PHQ-9, Patient Health Questionnaire 9; POD, postoperative day; postop, postoperative; preop, preoperative; PSQ, Pain Sensitivity Questionnaire; PSQI, Pittsburgh Sleep Quality Index; QoR-15, quality of recovery 15; QST, quantitative sensory testing; RAND36, RAND 36-item health survey questionnaire; REDCap, Research Electronic Data Capture; SET, sleep education therapy; THA, total hip arthroplasty; TKA, total knee arthroplasty; TSA, total shoulder arthroplasty; TSK, Tampa scale of Kinesiophobia; W, week(s); WOMAC OA, the Western Ontario and McMaster Universities Osteoarthritis Index.

Primary and secondary outcome measures are listed in online supplemental table 1. The timing of outcome assessment is shown in figure 1.

### Primary outcome

The primary outcome measure is:

- Average pain intensity (NRS 0–10) over the past 24 hours rated each morning, averaged over the first 7 postoperative days (PODs).

### Secondary outcome measures

#### Self-report

##### Brief pain inventory short-form

The brief pain inventory (BPI) generates two domain scores: mean pain severity score (based on four questions related to pain intensity, range 0–10; 0=no pain, 10=worst imaginable pain), and mean pain interference score (based on seven questions related to different dimensions of pain interference, range 0–10; 0=no pain interference, 10=complete pain interference).^37^

##### Western Ontario and McMaster Universities Osteoarthritis Index

The Western Ontario and McMaster Universities Osteoarthritis Index (WOMAC OA) consists of 24 items related to OA-related symptoms.^38^ Each item is scored 0–4 (0=none, 1=slight, 2=moderate, 3=severe, 4=extreme). Based on the 24 items, three subscores are created: pain (range, 0–20), stiffness (range, 0–8) and physical function (range, 0–68). Higher scores indicate worse symptomatology.

##### Prescription opioid use

Acute postoperative use of prescription opioid analgesics will be quantified on the first POD (POD1) through day 7 (POD 7) via self-report of number of doses consumed, using electronic chart review to verify opioid type and dose. At each follow-up time point, a 7-day Timeline Followback assessment^39^ will quantify past week daily opioid use. A total daily opioid use metric will be summarised as daily oral morphine milligram equivalents at each assessment occasion.

##### Quality of recovery 15

Recovery in the acute postoperative phase will be assessed through the quality of recovery 15 (QoR-15) questionnaire, administered on POD1–3 and POD7.^40^ The QoR-15 focuses on the last 24 hours and covers key domains related to recovery after surgery, for example, pain, sleep, physical comfort, physical independence, psychological support and emotional state. Each question is scored 0–10, generating a total score of 0–150 (0=very poor recovery, 150=excellent recovery). Two questions of the QoR-15 assess moderate and severe pain, respectively; the answer to these questions can be summarised as a total pain score ranging 0–20, with higher scores indicating lower pain intensity.

##### Insomnia Severity Index

The ISI is a brief, reliable and valid instrument to identify insomnia cases in population-based studies.^41^ Moreover, the ISI is sensitive to assessment of treatment response in clinical samples. The ISI consists of seven questions related to insomnia symptoms and the burden of insomnia. Each question is scored 0–4 which yields a total score of 0–28 (0–7: no clinically significant insomnia; 8–14: subthreshold insomnia; 15–21: clinical insomnia (moderate severity); 22–28: clinical insomnia (severe)).

##### Pittsburgh Sleep Quality Index

The Pittsburgh Sleep Quality Index (PSQI) is composed of 19 items, each scored 0–3 (no difficulty to severe difficulty); component scores are summed to create a global score, ranging from 0 to 21 (higher scores indicate worse sleep quality). A PSQI score >5 is often considered to demonstrate clinically significant sleep disturbance.^42^

##### Patient Health Questionnaire 9

The Patient Health Questionnaire 9 is a brief instrument assessing the nine DSM-IV criteria for depression.^43^ Each item is scored 0–3, generating a score range 0–27 with higher scores indicating more severe depressive symptoms (0–4: none-minimal depression severity; 5–9: mild; 10–14: moderate; 15–19: moderately severe depression severity; 20–27: severe depression).

##### Generalised Anxiety Disorder 7

The Generalised Anxiety Disorder 7 (GAD-7) is a valid, brief seven-item screening tool for generalised anxiety symptoms over the past 2 weeks.^44^ Scores range from 0 to 21, with higher scores being associated with more severe anxiety symptomatology and functional impairment; a score ≥8 is often used to identify probable cases of GAD, although cut-off scores 7–10 provide similar estimates of sensitivity/specificity.^45^

##### EuroQol 5 dimension 5 level

The EuroQol 5 dimension 5 level (EQ-5D-5L) has five dimensions: mobility, self-care, usual activities, pain/discomfort and anxiety/depression. Each dimension has five response levels. Additionally, the EQ-5D-5L includes a Visual Analogue Scale, which represents the subject’s self-rated health on a scale ranging between 0 and 100 (0=the worst health you can imagine, 100=the best health you can imagine). Single EQ-5D-5L index values can be obtained through an index value calculator using nation-level value sets.^46^

##### RAND-36

The RAND-36 is a widely used health survey consisting of 36 items which covers eight domains: physical functioning, bodily pain, role limitations due to physical health problems, role limitations due to personal or emotional problems, general mental health, social functioning, energy/fatigue and general health perceptions.^47^ Additionally, one item is included to assess perceived change in health over time. RAND-36 includes the same items as the short form 36 health questionnaire (SF-36), but the scoring algorithm differs slightly. Possible scores range from 0 to 100, with higher scores representing better health status. Normative data, generated through surveys of representative samples of the general population, are important for adequate interpretation of RAND-36 scores.^48^

### Objective measures

#### Actigraphic sleep continuity measures

To obtain objective measures of sleep continuity, for example, sleep onset latency, total sleep time, wakefulness after sleep onset and sleep efficiency (ie, total sleep time divided by time in bed), an actigraphic device worn on the wrist (MotionWatch 8, CamNtech, Manor Farm, Cambridgeshire, UK) will be used for several weeks throughout the study (see figure 1). Actigraphic devices record movements (and light) that can be used to estimate sleep parameters.^49 50^ Comparison to the gold standard for assessment of sleep, polysomnography, shows that actigraphy in general produces similar estimates of sleep duration (total sleep time) and sleep efficiency, although there may be an overestimation of these variables through actigraphic assessment in populations with poor sleep and low activity levels.^51^ Nevertheless, actigraphy provides the opportunity to assess sleep over extended periods in the person’s natural sleep environment, which is a great advantage compared with polysomnography, especially considering that insomnia symptoms are often variable over time.

#### Quantitative sensory testing measures of pain

To obtain assessments of pain thresholds and dynamic measures of pain (eg, central sensitisation), a comprehensive quantitative sensory testing (QST) protocol will be applied. All testing will be performed in a quiet, air-conditioned (20–24°C) room. Testing will be conducted by one out of two experienced research nurses who have obtained extensive training in the methods used. Throughout the QST, participants will be asked to keep their eyes closed to enable complete focus on the evoked sensations. Brief demonstrations of the different procedures will be performed prior to testing. The following procedures will be performed:

- Assessment of *fibromyalgia tender points* (according to American College of Rheumatology (ACR) 1990 criteria) through application of pressure (4 kg).
- Assessment of *pressure pain detection threshold* (PPDT) using a digital algometer (SBMEDIC Electronics, Solna, Sweden) with a 1 cm^2^ probe area applied to the skin at a constant rate of 30 kPa/second. The participant will be instructed to indicate (through pushing a button) when the stimulus ‘first feels painful’. Testing will be performed with the participant in a supine position. In the case of participants undergoing THA, four locations will be examined: (1) a proximal, volar area on the forearm (bilateral) and (2) the area overlying the major trochanter (bilateral). For those undergoing TKA, the locations will also include a proximal, volar forearm area, but instead of the major trochanter, an area adjacent (medial and proximal) to the femorotibial junction (ie, just above the ‘fatty pad’) will be examined (bilateral). Two trials with a 30 s interstimulus interval will be completed per area. Subsequently, pressure pain testing will be performed once in each area to ascertain which level of pressure (kPa) is required to elicit pain corresponding to NRS 4/10 (ie, PP4) and NRS 7/10 (ie, PP7), respectively.
- To examine *temporal summation* (TS), that is, central nervous system augmentation of pain caused by repeated painful stimulation, mechanical stimulation will be applied to three areas (THA: area overlying the major trochanter on the affected side, the contralateral area and ‘the triangle’ between the first and second metacarpal bones on the dorsum of the left hand; TKA: knee-area described above, the contralateral area and the same ‘triangle’ on the dorsum of the hand described above). A weighted pinprick stimulator (‘PinPrick’, MRC Systems GmBH, Heidelberg, Germany) calibrated to deliver 256 nM of force will first be applied to the skin in an area which will not be tested subsequently. The participant will be asked to rate how painful the stimulus is on a scale from 0 to 100 (0=no pain, 100=worst pain imaginable); if the participant perceives this stimulation to be painful, the 256 mN stimulator will be used throughout the TS testing, otherwise a 512 mN stimulator will be used. The participant will first rate how painful a single pinprick stimulus is in each of the three testing areas. Thereafter, a train of identical 10 pinprick stimuli, at a frequency of 1 per second (1 Hz), will be administered within an area of 1 cm^2^. The participant will rate the peak pain experienced during the testing sequence, whereafter TS will be calculated as peak pain rating divided by pain rating of single initial stimulus. In case the participant rates the first stimulus as 0 (ie, no pain), a minimum value of 1 will be assigned in order to enable calculation of TS. Assessment of TS will be performed twice in each area, with a 2 min pause between tests. Results from the two sets of testing will be averaged.
- *Conditioned pain modulation* (CPM), that is, the capacity of endogenous pain inhibitory systems, will be assessed through a QST paradigm comprising an ischaemic pain (tourniquet) conditioning stimulus and a pressure pain test stimulus (as described in detail by Tour *et al*^52^). First, PPDT will be assessed twice in a midpart area of the thigh contralateral to the hip/knee which will undergo surgery (near the crossing of the sartorius and rectus femoris muscles); subjects will also be asked to report pain intensity (NRS 0–100) in the contralateral arm. Then, a tourniquet will be placed on the contralateral arm, and subsequently inflated to constrict blood flow. The subject will then be given a 2 kg weight to perform reverse wrist curls, to elicit ischaemic pain in the left forearm and hand. Once the pain reaches a level corresponding to NRS 50/100, the investigator will repeatedly assess PPDT in the area specified above, at 30 s intervals. Maximally, eight measurements will be conducted prior to the release of the tourniquet. The subject will then be asked to rate the maximum arm pain perceived during the test, as well as pain in the hip/knee after the test. Finally, following 5 min of rest, two PPDT measurements will conclude the CPM testing session.
- To assess *cold pain tolerance*, a circulating, cold water bath (2°C) will be used (Somedic AB, Hörby, Sweden). Participants will first be asked to report pain intensity in their hip/knee and the contralateral hand. Thereafter, participants will immerse the contralateral hand up to the wrist in the cold water, up to an uninformed 4 min maximum time limit. Participants will be instructed to indicate when the cold first starts feeling painful (cold pain detection threshold), and to keep their hand in the water for as long as possible, until the cold pain becomes intolerable (cold pain tolerance threshold). If the participant is able to keep their hand in the water throughout the full 4 min period, the test will be aborted and the cold pain tolerance threshold assessed as 240 s. Immediately after the test, subjects will be asked about maximum pain in the hand, as well as pain in the hip/knee. Finally, after 5 min of rest, participants will once again be asked to report pain intensity in these locations.

#### Testing of neurocognitive function

Different aspects of neurocognitive function will be assessed through a comprehensive digital test battery (Mindmore application, Stockholm, Sweden)^53 54^ including:

- Rey auditory learning test—captures the ability to encode, store and recover verbal information.
- Trail-making test—assesses attention and processing speed
- Corsi block-tapping test—assesses visuospatial short-term and working memory
- Stroop colour and word test—assesses the ability to inhibit cognitive interference
- Symbol digit processing test—assesses information processing speed
- Paced auditory serial addition test—examines attention and working memory.

#### Assessment of blood biomarkers

Blood samples (approximately 24 mL per participant) will be collected by venipuncture. To characterise changes in markers which are relevant to pathways involved in pain and sleep processes, several analyses are planned, including assessment of inflammatory mediators (eg, via Olink proximity extension assay technology), coagulation, monoamine metabolites (through high-performance liquid chromatography), large-scale proteomic analyses, as well as assessment of relevant lipids, metabolites and small molecules (via mass spectrometry).

### Other prespecified outcome measures

#### International Physical Activity Questionnaire short form

The International Physical Activity Questionnaire short form will be used to assess subjective activity level.^55^ Separate scores are generated for walking, moderate-intensity and vigorous-intensity activities. Both categorical (inactive, minimally active, health-enhancing physical activity active) and continuous (median metabolic equivalent of task (MET)-minutes per week; MET=multiple of the resting metabolic rate) measures of physical activity can be calculated. Kilocalories can be computed from MET-minutes.

#### Pain Catastrophising Scale

The Pain Catastrophising Scale (PCS) will be used to identify clinically significant pain catastrophising, that is, negative pain-related thoughts and distress (often described in three domains: rumination, magnification and helplessness).^56^ The PCS contains 13 items, each scored 0–4, which yields a total score ranging between 0 and 52, with higher scores indicating more pain catastrophising thoughts. A total score ≥30 suggests clinically relevant levels of catastrophising.

#### Tampa Scale for Kinesiophobia 17

The Tampa Scale for Kinesiophobia 17 provides a valid and reliable subjective rating of kinesiophobia (ie, an irrational and debilitating fear of physical movement and activity), based on 17 items.^57^ Each item consists of a statement that is linked to fear of movement, reinjury or avoidance, which the user rates on a 4-point Likert scale (strongly disagree—disagree—agree—strongly agree). Scores range from 17 to 68; higher scores indicate an increasing degree of kinesiophobia, with scores above 37 often considered to show significant fear of movement.^58^ In addition, two subscale scores are often derived (activity avoidance, centred around the belief that activity may be harmful or increase pain; and somatic focus, which reflects the belief in underlying, serious medical problems).

#### Pain Sensitivity Questionnaire

The Pain Sensitivity Questionnaire (PSQ) is a self-rating measure for pain perception which consists of 14 items describing imagined painful situations in daily life.^59^ Additionally, for baseline reference, three normally non-painful situations are interspersed among the items. The items assess different pain modalities, for example, thermal, chemical and mechanical noxious stimuli, across various body sites and are rated on an NRS from 1 (not painful at all) to 10 (worst pain imaginable). Based on the items, a total score (average rating of all 14 items) and two subscores are generated, PSQ moderate and PSQ minor (each based on average rating of 7 different items). Previous studies have shown that PSQ scores are associated with experimentally obtained pain intensity ratings and pain behaviour in healthy subjects and pain patients.^60 61^

#### ACR 2016

The ‘ACR 2010/2011’ fibromyalgia criteria^62^ have been slightly revised in 2016, and are therefore often referred to as the ‘ACR 2016 criteria’.^63^ They consist of two subscales, the Widespread Pain Index and the Symptom Severity Scale, the sum of these is referred to as the fibromyalgia severity score (FS) and has frequently been used to assess the degree of ‘fibromyalgianess’, that is, the degree of nociplastic pain components. The FS score has been identified as a robust predictor of poorer outcome following TKA and THA and a predictor of higher opioid consumption following surgery, also in individuals who do not fulfil the criteria for fibromyalgia.^64 65^

#### Chronic Pain Acceptance Questionnaire

The Chronic Pain Acceptance Questionnaire-8^66^ will be used to assess pain-related acceptance, which entails a willingness to pursue valued activities regardless of pain.^67^ The measure consists of eight items arranged on two subscales: pain willingness and activity engagement. Each item is rated on a scale from 0 to 8, yielding a full score ranging from 0 to 48. Higher scores reflect greater pain acceptance, which is associated with lower levels of psychological distress and pain interference among individuals with chronic pain.^66 68 69^

### Participant timeline

An overview of the timeline and workflow of study procedures is provided in figure 1 and online supplemental table 2.

#### The first preoperative phase of the study

Potential study participants will be identified through screening of the digital surgery planning software Orbit. Patients who are scheduled to undergo TKA or THA at the Elisabeth Hospital in Uppsala or the orthopaedic surgical department in Enköping will be given study information documents and an invitation to complete brief, initial screening questions, including the ISI. Patients who report sleep problems corresponding to ISI score >10, that is, clinical insomnia, will be contacted by telephone for more in-depth eligibility screening, including confirmation of insomnia disorder according to DSM-V criteria, and subsequently invited to a first onsite visit to the multidisciplinary pain clinic at Uppsala University Hospital, at least 8 weeks prior to surgery. During visit 1, final check of inclusion and exclusion criteria will be performed, whereafter eligible participants who decide to enter the study will provide written consent.

As shown in figure 1 and online supplemental table 2, procedures during visit 1 entail completion of questionnaires, QST, digital cognitive testing, venipuncture to obtain blood samples and initiation of actigraphy. When visit 1 has been completed, participants will be randomised to either hybrid-format CBT-I treatment or the active comparator control, SET. The intervention will be carried out over the course of 4 weeks, according to the detailed description above, in a hybrid format including digital CBT-I or SET with addition of telehealth video-sessions with a psychologist (CBT-I) or research nurse (SET).

#### The second preoperative phase of the study

When the preoperative phase of the intervention has been completed, participants will undergo a second onsite assessment, at least 4 weeks before surgery, to repeat almost all study procedures which were administered during visit 1. Actigraphy data will be downloaded, whereafter actigraphs will once again be provided to participants to collect data up until 2 weeks postoperative. Hence, objective sleep continuity data across the whole perioperative period will be obtained.

#### The postoperative phase of the study

Participants will complete questionnaires, through the REDCap (Research Electronic Data Capture)^70 71^ electronic data capture tool hosted at Uppsala University, during the first 7 PODs, to assess the acute postoperative pain trajectory, opioid consumption and recovery parameters, for example, physical function, nausea, sleep, anxiety and depression symptoms. Two weeks postoperative, actigraphs will be returned through mail. As described above, a telehealth CBT-I or SET booster session will be administered 1–2 weeks after surgery. 3 months postoperative, participants will complete a small number of questionnaires (BPI, WOMAC OA, ISI). 6 months postoperative, participants will complete the third and final on-site visit, to repeat the procedures which were performed during visit 2, with addition of testing for CPSP according to International Association for the Study of Pain (IASP) criteria. The final data collection will take place 12 months postoperative, using the same questionnaires as for the remote 3-month follow-up assessment.

### Recruitment and retention

Based on the latest annual report from the Swedish joint arthroplasty registry, our own data^72^ and the present inclusion/exclusion criteria, we can assume that two persons undergoing THA or TKA at the two operating sites might be eligible for inclusion per week. Based on our power calculation, we anticipate that the full study population could be recruited in 2–3 years. Nevertheless, recruitment to the intervention may prove challenging in the context of major orthopaedic surgery with postoperative symptom burden and substantial energy directed towards physical therapy, etc. If recruitment proves to be complicated, efforts can be made to expand inclusion to additional nearby elective orthopaedic surgical centres. Prior to the addition of any surgical centres, amendment application(s) for approval by the Swedish Ethical Review Authority will be submitted. Additionally, the mode of invitation to take part in the study, currently through a single letter, could be altered to promote recruitment. Such strategies might include reminder letters, phone calls or enhanced promotion of the study already at the first orthopaedic preoperative appointment (when the surgery is scheduled). To compensate for travel expenses and time devoted to the study, all participants will receive Kr1000 per in-person visit (ie, a total of Kr3000 for completion of three visits). The pace of recruitment will be closely monitored throughout the trial. To enhance retention, text messages will be sent, and phone calls made, to confirm dates of follow-up assessments, both for on-site visits and remote assessments. Reminders will be sent to participants who fail to reply to follow-up invitations. If a participant finds it too burdensome to complete all questionnaires or procedures at one or more follow-up assessments, the participant will be given the opportunity to continue with completion of a reduced protocol, maintaining key outcomes. Reasons for non-adherence to the interventions will be recorded.

### Randomisation sequence generation

The allocation sequence will be created via a computerised random number generator with equal probability to the interventions (1:1) within blocks by surgery type. The randomisation procedure will ensure that equal proportions of participants undergoing THA and TKA, respectively, are assigned to the two interventions.

### Concealment mechanism (allocation concealment)

Allocation concealment will be ensured using sequentially numbered, opaque, sealed envelopes, until the patient has been recruited into the trial, which takes place after the baseline assessment (visit 1) has been completed.

### Blinding (masking)

To reduce the risk of bias, investigators, outcome assessors and data analysts will be unaware (blind) of the intervention to which the trial participants have been assigned, on intervention assignment and beyond. Participants will be aware that they are assigned to an intervention for the treatment of insomnia. The consent form states that participants will be assigned at random to either CBT-I or SET, and that both interventions are used for treatment of insomnia. The programme content of the alternative treatment will not be revealed. Although there is limited information related to objectives of the trial in the consent form, participants will be blind to most objectives and study hypotheses.

### Data collection methods and data management

Data will be collected through REDCap, in paper forms (eg, some questionnaires, interviews, QST results) and in digital format (eg, cognitive testing, actigraphic assessment). Data will be transcribed from source documentation directly into a statistical programme (eg, SPSS), or, in the case of results obtained through digital cognitive testing, directly captured and converted into a statistical programme. When data are collected on paper forms, data entry will be performed locally, to enable immediate correction of missing or inaccurate data. Assessors (research nurses) will receive training to promote data quality. Prior to analyses, the lead statistician will verify that the data are in the proper format and within an expected range of values. Additionally, there will be an independent verification of a random subset of data (∼10%) to identify missing or erroneous values. During, and up until 10 years after the trial has been completed, paper forms will be stored securely in binders placed in locked file cabinets at the study site.

### Statistical methods

A comprehensive statistical analysis plan will be approved before any analyses are conducted. All analyses will be reported according to the CONSORT (Consolidated Standards of Reporting Trials) statement.^73^ The primary analyses will focus on intent-to-treat samples, but per-protocol analysis based on treatment exposure (ie, insomnia treatment compliance) will be considered as secondary. All measured variables will be assessed for distributional qualities and transformed if necessary for use in the selected statistical models. IBM SPSS V.29 will be used for the majority of analyses, though other common validated statistical software packages may also be used.

The basic design is a parallel group (Patient Group: TKA and THA), two arm (Treatment Condition: CBT-I vs education control), independent balanced analysis of variance with one or more repeated outcome measures (Time) analysed with linear mixed models (LMMs). The number of repeated measures varies by assessment; for the primary analysis of NRS scores, the individual scores will be modelled rather than calculating and comparing averages over the time points. LMMs have the advantage of using all available data to generate unbiased estimates, assuming any missing data meets the definition of missing at random. The key results are the main effects of Treatment Condition and their interaction over Time. The interactions of Patient Group with the Treatment Condition and/or with Time are certainly of interest but secondary; specifically, we are most focused on the impact of the treatment across patient groups rather than variations between patient groups at this stage of the research. However, Patient Group will be a covariate in primary analyses.

The sample size calculations are focused within the primary analyses. Specifically, the key outcomes are the changes from baseline (Visit 1) in the pain measures to post-treatment (Visit 2) and immediate postsurgery (POD1–7). Generalized linear model (GLM) simulations using data ranges based on published studies^1 28 74^ to estimate the effect sizes of the CBT-I treatment on insomnia (medium to large)^28 75^ and the impact of the reduction of insomnia on measures of pain (medium)^1 74^ suggest that samples of 63–88 are required for 80% power (α=0.05, two-tailed); these simulations considered various combinations of effects that represented a range of small (d=0.25) to medium (d=0.55) effects over the eight key outcome assessments. A total sample size n=100 (TKA, THA combined) was selected given the upper limit and some loss of follow-up. The estimated effect size is smaller than a two-point change in the NRS (0–10) which is commonly considered the minimal clinically important difference for acute postoperative pain intensity after THA or TKA.^76^ Secondary analyses examining the additional variables and time points will be identical in execution; however, the impact of CBT-I may be reduced with lower statistical power.

### Monitoring of data and safety

Given known minimal risks associated with the interventions used, there is no need for a formal safety monitoring committee or interim analysis. To evaluate the progress of the trial and identify potential problems, the principal investigator will designate appropriately qualified personnel to periodically perform quality assurance checks. The monitor will assure that data are accurate and in agreement with any paper source documentation used, verify that participants’ written consent has been properly obtained and documented, confirm that participants entered into the study meet inclusion and exclusion criteria, verify that study procedures are being conducted according to the protocol guidelines and review any adverse events. Specifically, given that sleep restriction and stimulus control have been associated with initial increased sleepiness and fatigue, these symptoms will be assessed as part of routine monitoring. Meetings with an independent data and safety monitoring board will be scheduled if there are any serious adverse events or harms that might be considered at least possibly related to the study procedures. Adverse events will be collected after the participant has provided consent, enrolled in the study and started the intervention.

### Ethics and dissemination

The Swedish Ethical Review Authority has approved the PROSAP-A trial protocol (Dnr 2023-03976-01). Results will be published in international peer-reviewed journals and summaries will be provided to funders and participants of the trial.

### Consent

All potential participants of the study will be given a copy of the study information and informed consent document when they are first invited to take part in the study, as part of the invitation letter package. Potential participants will then have ample opportunity to ask questions both in written form (through e-mail), over phone (during screening) and in person (during visit 1, prior to signing the consent form). All aspects of the study and informed consent will be explained in lay language to the participant by an experienced research nurse. Any person who is unable to demonstrate understanding of the information contained in the informed consent will be excluded from study participation.

### Protocol amendments

Prior to protocol amendment, any substantial protocol changes will be submitted to the Swedish ethical review authority for approval, by the principal investigator. Moreover, changes to the study protocol (eg, eligibility criteria, outcomes, analyses) will be submitted by the principal investigator to update the online study registration records at ClinicalTrials.gov (NCT06145516).

### Confidentiality

All data in the study will be pseudonymised, that is, participants are identified by a unique trial ID code (an unrelated sequence of characters and numbers). All paper source documents will be marked by this ID code and stored in locked file cabinets in a room with limited access at the study site (Multidisciplinary Pain Center, Uppsala University Hospital, Sweden). Personal information about potential and enrolled participants will be stored and maintained using encrypted digital files within password-protected folders on a computer with adequate firewall protection, stored securely at the study site. All blood samples will be identified by a coded ID number to maintain participant confidentiality. No personal identifier will be used in any publication or communication used to support this trial. Access to study-related documents will be limited to the minimum number of individuals necessary to ensure quality, safety and effective execution of trial procedures.

### Access to data

The principal investigator and the lead statistician will have access to the final trial data set. To ensure confidentiality, any partial data sets dispersed to project team members for analysis will be blinded of any identifying participant information (pseudonymised).

### Patient and public involvement

Patients or the public were not involved in the design, conduct, reporting or dissemination plans of this protocol.

## Discussion

To date, only a very limited number of studies have evaluated perioperative sleep interventions. No previous study has implemented a comprehensive preoperative psychological intervention, such as CBT-I, to achieve improved perioperative sleep quality and quantity, with the potential to positively impact both acute measures of pain and recovery, as well as incremental effects on multiple long-term postoperative health outcomes. The populations which the study targets, patients with persistent knee or hip osteoarthritis-related pain, suffer severely due to both sleep disturbance and pain symptoms. The intervention has potential to enhance both the physical and mental health benefits that are often obtained through arthroplasty surgery.

The study has numerous methodological strengths and innovations that have the potential to meaningfully advance our understanding of the interrelations of sleep and pain. First, it targets sleep in the preoperative period prior to orthopaedic surgery, adapting the ‘prehabilitation’ model that has demonstrated clinical effectiveness with physical therapy.^77^ This approach addresses *both* preoperative sleep and pain dynamics in patients with chronic pain preparing for a joint replacement surgery *and* postoperative sleep and pain needs directly stemming from surgery. As surgery can compound pre-existing sleep and pain problems, the prehabilitation treatment model (including postoperative booster sessions) employed in the proposed study will address sleep and pain associations as they occur throughout the spectrum of perioperative care. It is notable that pain is an ‘off-target’ outcome of an intervention designed explicitly to improve sleep. Although ample experimental and longitudinal evidence now indicates that sleep disturbance prospectively predicts increased pain severity in chronic pain populations,^4 78 79^ trials of CBT-I in patients with chronic pain have resulted in weak and variable effect sizes on pain outcomes.^23 80^ Our study, however, will evaluate pain changes following CBT-I in a novel clinical context (recovery from hip or knee surgery) that may foster unique effects. Given the centrality of sleep to surgical recovery and the well-defined postoperative recovery period, there is a strong theoretical and methodological basis for hypothesising that sleep improvements following CBT-I will yield pain reductions in the present study. This contrasts with studies of CBT-I in chronic pain, in which sleep and pain changes are typically evaluated in samples composed of individuals who have experienced chronic pain in time arcs spanning months to decades.

Second, this study employs a wide array of multimodal assessments that will facilitate discovery of novel mechanisms of the association of sleep and pain. As sleep disturbance degrades the functioning of multiple organ systems and contributes to a distributed pathophysiology, it will be important to evaluate multiple putative pathways leading from preoperative sleep disturbance (and sleep restoration via CBT-I) to postoperative pain (and functional restoration). The proposed blood sampling will enable testing of inflammatory, monoaminergic and genetic mediators of the association of sleep and pain, whether they respond to CBT-I, and whether such responses predict postoperative pain and functional trajectories out to 1-year follow-up. The comprehensive QST battery will index individual variability in pain sensitivity as well as descending pain facilitation and inhibition. These assessments will complement the clinical pain assessments and shed light on the role of endogenous pain modulatory processes in the evaluation of perioperative sleep and pain. Further, the neurocognitive performance assessment will provide critical insights into the potential relationship between sleep and perioperative cognitive changes, such as postoperative delirium, which are common^81^ and influenced by perioperative pain and analgesic efficacy.^82^,^84^

Third, orthopaedic surgery is among the most common clinical settings for outpatient opioid prescriptions, which can extend into long-term opioid therapy.^85 86^ As both sleep disturbance^87 88^ and pain^89 90^ are upstream predictors of risk for problematic opioid use, the proposed study has the potential to meaningfully contribute to the widespread efforts underway to prevent opioid use disorder and opioid overdose.^91^ The longitudinal nature of the study design will permit an evaluation of whether long-term prescription opioid use can be mitigated by CBT-I effects on sleep and pain in the perioperative period. The importance of perioperative sleep disturbance, as a modifiable risk factor for multiple negative postoperative outcomes and complications, has previously been given limited attention. Further taking advantage of the longitudinal study design, repeated, objective measures of sleep continuity and detailed pain phenotyping will enable analyses that can characterise mechanisms and factors which can explain how sleep and pain relations evolve over time, and in detail map how sleep influences different outcomes during the perioperative phase.

Given the breadth and frequency of assessments, participant burden is an important consideration. We will, therefore, query participants early in the trial to determine if the volume of assessments is tolerable and the burden balanced against the potential benefits of treatment. If the study is deemed overly burdensome, we will adjust by paring down secondary outcome assessments as needed, without compromising the focus on primary outcomes.

Additionally, although internet and internet-enabled devices are highly accessible in Sweden, we will work with prospective participants to ensure there is equitable access and fluency across the sample. If eligible participants lack the proper access or fluency, we will provide the necessary devices and/or training to facilitate participation in the digital CBT-I intervention, telehealth visits and online data collection.

## Supplementary material

10.1136/bmjopen-2025-099785 online supplemental file 1

10.1136/bmjopen-2025-099785 online supplemental file 2
