# Supplementary material for: Randomised, controlled clinical trial evaluating the effects of preoperative insomnia treatment on postoperative pain control and recovery: a protocol for the Promoting Sleep to Alleviate Pain-Arthroplasty (PROSAP-A) trial
Source: BMJ Open. 2025 Jul 30;15(7):e099785. doi: 10.1136/bmjopen-2025-099785 (PMC12314951; doi:10.1136/bmjopen-2025-099785)
Supplement: online supplemental file 1 [file bmjopen-15-7-s001.pptx]

## Slide 1
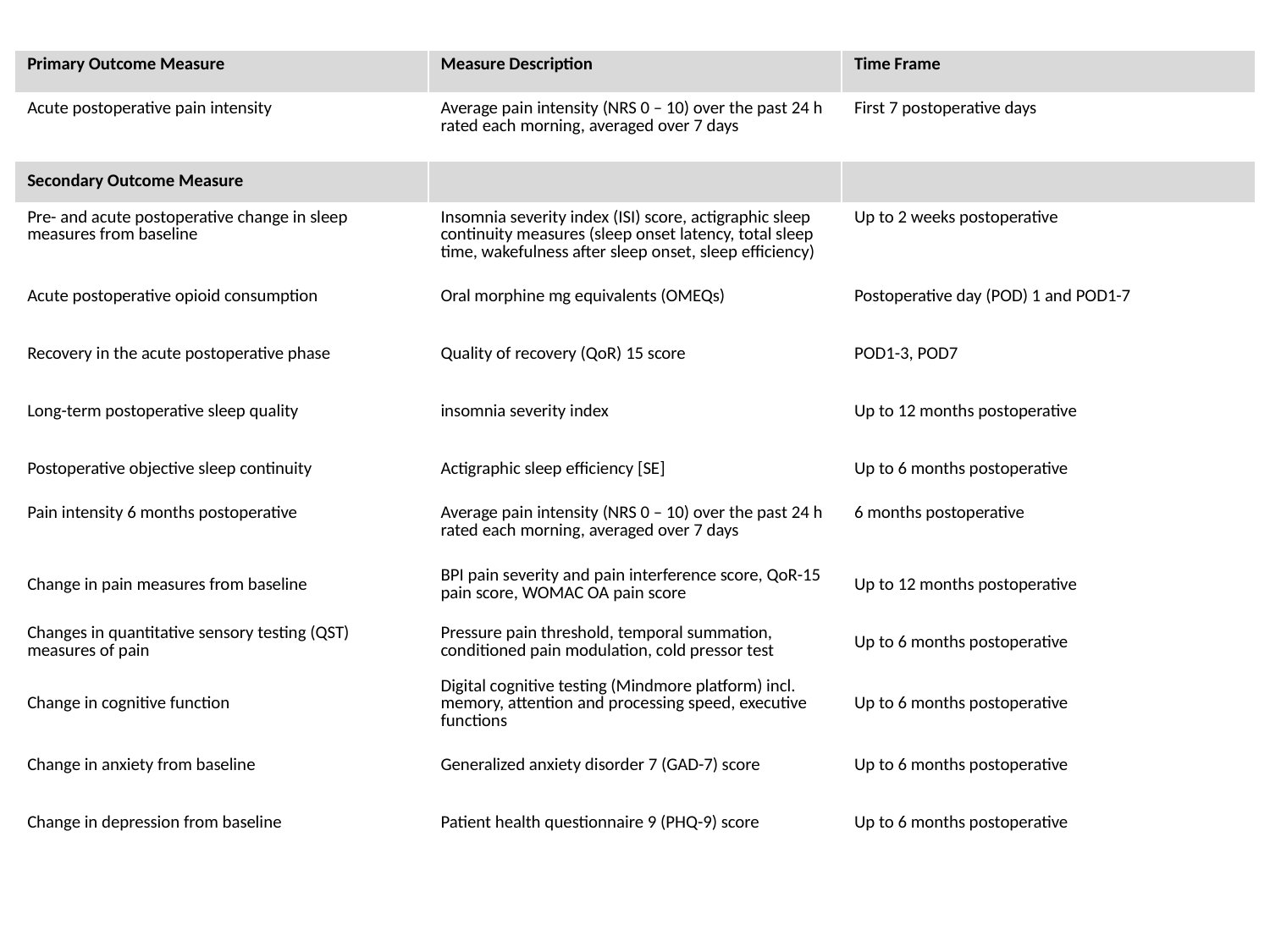

| Primary Outcome Measure | Measure Description | Time Frame |
| --- | --- | --- |
| Acute postoperative pain intensity | Average pain intensity (NRS 0 – 10) over the past 24 h rated each morning, averaged over 7 days | First 7 postoperative days |
| Secondary Outcome Measure | | |
| Pre- and acute postoperative change in sleep measures from baseline | Insomnia severity index (ISI) score, actigraphic sleep continuity measures (sleep onset latency, total sleep time, wakefulness after sleep onset, sleep efficiency) | Up to 2 weeks postoperative |
| Acute postoperative opioid consumption | Oral morphine mg equivalents (OMEQs) | Postoperative day (POD) 1 and POD1-7 |
| Recovery in the acute postoperative phase | Quality of recovery (QoR) 15 score | POD1-3, POD7 |
| Long-term postoperative sleep quality | insomnia severity index | Up to 12 months postoperative |
| Postoperative objective sleep continuity | Actigraphic sleep efficiency [SE] | Up to 6 months postoperative |
| Pain intensity 6 months postoperative | Average pain intensity (NRS 0 – 10) over the past 24 h rated each morning, averaged over 7 days | 6 months postoperative |
| Change in pain measures from baseline | BPI pain severity and pain interference score, QoR-15 pain score, WOMAC OA pain score | Up to 12 months postoperative |
| Changes in quantitative sensory testing (QST) measures of pain | Pressure pain threshold, temporal summation, conditioned pain modulation, cold pressor test | Up to 6 months postoperative |
| Change in cognitive function | Digital cognitive testing (Mindmore platform) incl. memory, attention and processing speed, executive functions | Up to 6 months postoperative |
| Change in anxiety from baseline | Generalized anxiety disorder 7 (GAD-7) score | Up to 6 months postoperative |
| Change in depression from baseline | Patient health questionnaire 9 (PHQ-9) score | Up to 6 months postoperative |

## Slide 2
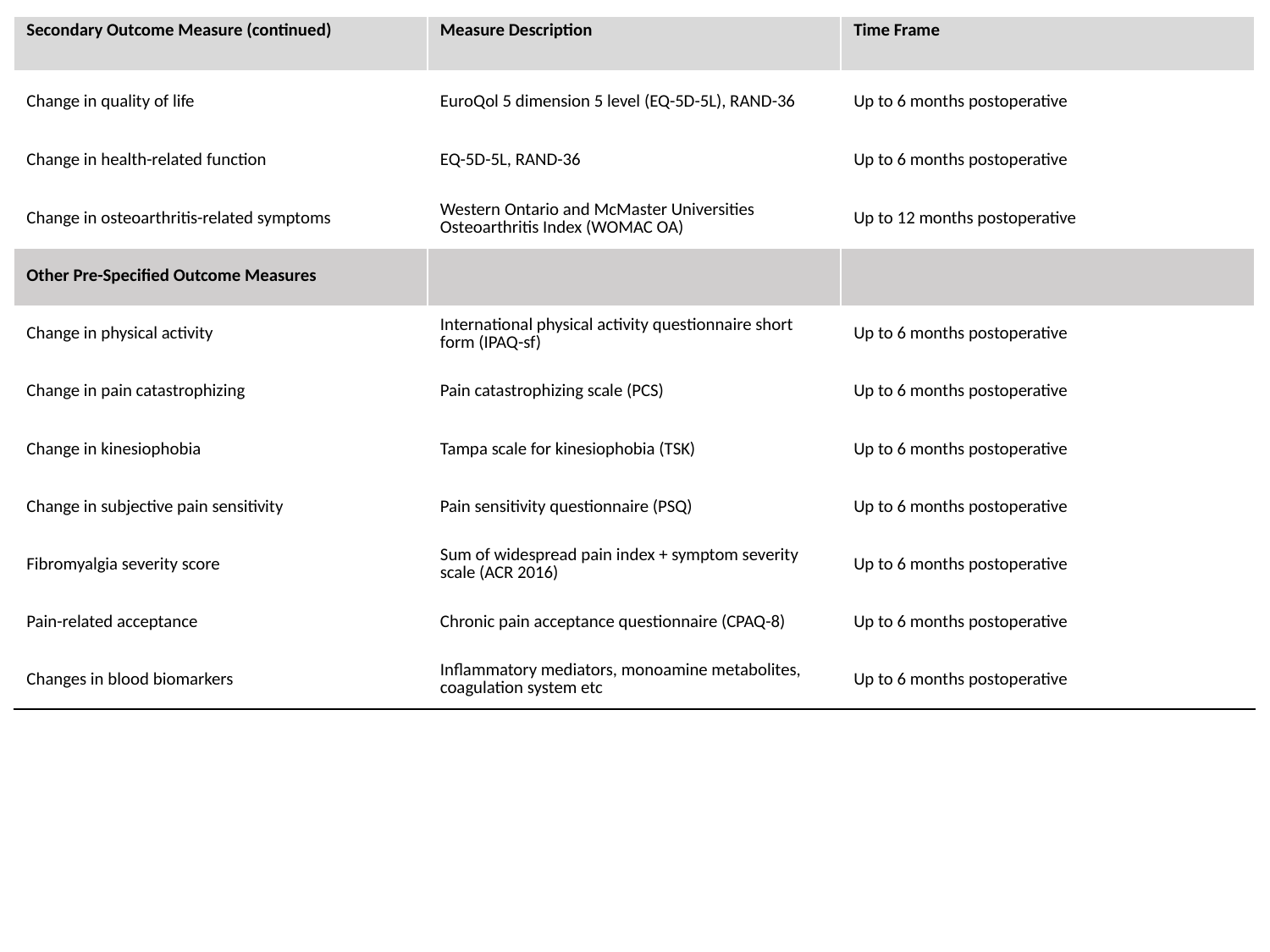

| Secondary Outcome Measure (continued) | Measure Description | Time Frame |
| --- | --- | --- |
| Change in quality of life | EuroQol 5 dimension 5 level (EQ-5D-5L), RAND-36 | Up to 6 months postoperative |
| Change in health-related function | EQ-5D-5L, RAND-36 | Up to 6 months postoperative |
| Change in osteoarthritis-related symptoms | Western Ontario and McMaster Universities Osteoarthritis Index (WOMAC OA) | Up to 12 months postoperative |
| Other Pre-Specified Outcome Measures | | |
| Change in physical activity | International physical activity questionnaire short form (IPAQ-sf) | Up to 6 months postoperative |
| Change in pain catastrophizing | Pain catastrophizing scale (PCS) | Up to 6 months postoperative |
| Change in kinesiophobia | Tampa scale for kinesiophobia (TSK) | Up to 6 months postoperative |
| Change in subjective pain sensitivity | Pain sensitivity questionnaire (PSQ) | Up to 6 months postoperative |
| Fibromyalgia severity score | Sum of widespread pain index + symptom severity scale (ACR 2016) | Up to 6 months postoperative |
| Pain-related acceptance | Chronic pain acceptance questionnaire (CPAQ-8) | Up to 6 months postoperative |
| Changes in blood biomarkers | Inflammatory mediators, monoamine metabolites, coagulation system etc | Up to 6 months postoperative |
