# Supplementary material for: Randomised, controlled clinical trial evaluating the effects of preoperative insomnia treatment on postoperative pain control and recovery: a protocol for the Promoting Sleep to Alleviate Pain-Arthroplasty (PROSAP-A) trial
Source: BMJ Open. 2025 Jul 30;15(7):e099785. doi: 10.1136/bmjopen-2025-099785 (PMC12314951; doi:10.1136/bmjopen-2025-099785)
Supplement: online supplemental file 2 [file bmjopen-15-7-s002.pdf]

			-2	-1	BL	1	2	3	4	5	6	7	8	9	10	11	12
**Activity/ assessment**	**Staff member**	**Approx. duration**	**Pre-study #1**	**Pre-study #2**	**BL visit**	**Tele-health #1**	**Tele-health #2**	**Tele-health #3**	**Tele-health #4**	**Visit 2 (preop)**	**FU POD7**	**Receive actigraph**	**Intervention booster session**	**FU 3M**	**Visit 3 (6M postop)**	**Receive actigraph**	**FU 12M**
Invitation letter	RN	10 min	X														
Telephone screening	RN	15 min		X													
Consent	RN	5 min			X												
Q´naires	RN *	15 min			X					X	X			X	X		X
QST	RN	45 min			X					X					X		
Cognitive testing	RN *	30 min			X					X					X		
Blood sampling	RN	10 min			X					X					X		
Initiate actigraphy	RN	5 min			X					X					X		
Download actigraphy	RN	5 min								X		X				X	
Randomize	RN	5 min			X												
Initiate digital intervention	PSY	15 min			X												
Video intervention	RN / PSY	30-45 min				X	X	X	X				X				
SAEs form	RN	N/A			As needed throughout protocol												
DSM-V criteria	RN	5 min								X					X		
MyCap invitation	RN	5 min								X					X		
