# Supplementary material for: Randomised, controlled clinical trial evaluating the effects of preoperative insomnia treatment on postoperative pain control and recovery: a protocol for the Promoting Sleep to Alleviate Pain-Arthroplasty (PROSAP-A) trial
Source: BMJ Open. 2025 Jul 30;15(7):e099785. doi: 10.1136/bmjopen-2025-099785 (PMC12314951; doi:10.1136/bmjopen-2025-099785)
Supplement: Supplementary file 3 [file bmjopen-2025-099785.reviewer_comments.pdf]

PEER REVIEW HISTORY

BMJ Open publishes all reviews undertaken for accepted manuscripts. Reviewers are asked to complete a checklist review form and are provided with free text boxes to elaborate on their assessment. These free text comments are reproduced below.

ARTICLE DETAILS

Title (Provisional)

A Randomized, Controlled Clinical Trial Evaluating the Effects of Preoperative Insomnia Treatment on Postoperative Pain Control and Recovery: A Protocol for the Promoting Sleep to Alleviate Pain - Arthroplasty (PROSAP-A) Trial

Authors

Bjurström, Martin F.; Bothelius, Kristoffer; Maathz, Pernilla; Jernelov, Susanna; Kraepelien, Martin; Rosenström, Alexander H.C.; Niklasson, Andrea; Smith, Michael. T.; Olmstead, Richard; Irwin, Michael R.; Finan, Patrick H.; Kosek, Eva

---

VERSION 1 - REVIEW

**Reviewer**	**1**
**Name**	**Simpson, Melanie R.**
**Affiliation**	**Norwegian University of Science and Technology, Department of Public Health and Nursing**
**Date**	**12-Mar-2025**
**COI**	**None**

Bjurström and co-authors describe an interesting and well planned randomised control trial that seeks to assess the effect of cognitive behavioural therapy for insomnia (CBT-I) on postoperative pain control in the first days postoperatively and 6-months after total knee arthroplasty or total hip arthroplasty. The secondary outcomes include a large range of assessments ranging from changes in quantitative sensory testing variables (e.g., temporal summation, conditioned pain modulation), sleep, cognition (e.g., attention, memory, processing speed, executive function), mental health, health-related function, physical activity, quality of life, and blood biomarkers.

The rationale and general design of the trial are clear and suitable. My main comments are regarding clarifications around the randomisation, sample size calculations and statistical analysis strategies.

1. Randomisation: It is described that the randomisation procedure will ensure equal proportions of participants undergoing THA and TKA. I have interpreted this to mean that the randomisation will be stratified by surgical procedure (later described as "Patient

Group"). Is this correct? Will there be (or is there) block randomisation to ensure ongoing balance in numbers? This is not necessary, but the description implies a greater control of distribution of THA and TKA participants than a simple randomisation may end up giving, so it would be good to clarify.

2. It is unlear how many sites are included in the study. Under "Study setting" it is described that surgeries will be performed at the Elisabeth hospital Uppsala or Enköping hospital, but also that "All three involved hospitals are in urban settings". Is the third hospital the Pain Center at Uppsala University Hospital where patients from both of the the surgery-performing hospital will be followed up? If there are multiple sites, will / is the randomisation stratified by site.

3. Secondary outcomes: there are many secondary outcomes described, and many of them have subscales, or can be analysed both as continuous scales or with previously defined cuf-offs. If the research already considers some of these outcomes to be of greater interest / importance than others, I recommend more clearly defining the key secondary outcomes and what are considered more exploratory.

4. General comment on statistical methods: With two primary outcome points, many secondary outcomes, a number of choices required in the analysis strategy, it is advisable to write a "Statistical Analysis Plan" prior to analysis and to be finalised before unblinding of the dataset. This is a requirement for some journals. See: https://www.equator-network.org/reporting-guidelines/guidelines-for-the-content-of-statistical-analysis-plans-in-clinical-trials/

5. Statistical methods, second paragraph: The following description is unclear - "The basic design is a 2 Treatment Condition (CBT-I vs education control) by 2 Patient Group (TKA and THA), independent balanced analysis of variance (ANOVA) with one or more repeated outcome measures (Time) analyzed with linear mixed models (LMM)." Firstly, the 2 x 2 design is usually used to describe a trial with two randomly assigned treatment conditions. Thankfully you are not randomising surgical procedure, so the description of a two-arm, parallel-group design which is found in the Abstract seems more correct. As stated the main effect of interest is that of treatment condition, with a secondary plan to investigate the modifying effect of surgical procedure on the effect of treatment. The second unclear aspect of the sentence above is that it implies that ANOVA is a part of the design of the trial, whereas both ANOVA (with repeated measures) and LMM are both analysis strategies. Based on the rest of the paragraph, it becomes clear that LMM is the planned analysis strategy, but remains unclear whether the use of ANOVA is also planned.

6. For the primary analyses, it would be interesting to have a more detailed description of the LMM model - For example, will daily pain scores be included, or an average across each of the two 7-day periods? Will the model include a main effect of treatment, or estimate a common baseline and only post-randomisation differences between treatment groups (as recommended in Twisk et al 2018:

https://www.sciencedirect.com/science/article/pii/S2451865417301849)? Surgery type will be included as a covariate, will other covariates be considered?

7. Sample size calculation / power calculations (last paragraph in Statistical methods): Generally, this paragraph could be described more clearly:

a) It is unclear what is meant by "The lower limits of statistical power are focused within the primary analyses". Does this mean that the primary outcomes were used to calculate required sample size based on a minimum desired statistical power?

b) The [primary] outcomes described in this paragraph are sleep and pain at visit 2, post-op day 1-7 and 7-day periods at 3, 6 and 12 months. The methods section otherwise implies that only pain on day 1-7 and at 6 months are primary outcomes. Were both sleep and pain and all follow-up time points considered in sample size calculations, or only some of these?

c) Was any adjustment made for the use of two primary outcomes (pain at day 1-7 and pain recorded over 7-days at 6 months)

d) Simulations seem like a very good strategy with this complex dataset, but it is unclear what level of pain reduction was considered to be clinically important in the calculations / what was the effect size the calculations were based on. Was the effect size in the sample size calculations defined based on findings from previous studies, and were these findings on what is clinically important or what CBT-I has previously achieved of difference in pain-scores? If the sample size is based on previously achieved differences in pain scores after CBT-I, are these clinically significant differences? If the sample size is based on MSD, can these be related to expected differences based on baseline pain scores and standard deviations?

e) Is the required sample size n=100 per group, or total?

f) It is realistic that only 12 of 100 participants are lost to follow-up?

g) The following statement is unclear: "As the groups will be balanced by Patient Group, all main effects and interactions of this factor will all be tested with equal, maximized power for the study design." This also implies that the randomisation strategy will achieve balance (see comment on stratification and block randomisation above). I am unsure if including an interaction term between treatment group and surgical procedure (i.e. Patient Group) will provide greater statistical power in the estimation of the main effect of treatment. I would be interested in a reference for this statement.

h) Will the described "Within Patient Group analyses" aim to estimate the effect of surgical procedure on pain level, or the modifying effect of surgical procedure on the effect of treatment on pain level.

Reviewer 2

Name **Thorlund, Jonas**

Affiliation **University of Southern Denmark, Institute of Sports Science and Clinical Biomechanics**

Date **10-Apr-2025**

COI **None**

Thank you for the opportunity to review this well-written and detailed protocol for a trial comparing CBTI with sleep education for postoperative pain in patients undergoing THA or TKA for osteoarthritis. I think this is a very relevant study,

Given the study is already ongoing my comments are mainly related to improving the description of the study in the protocol.

Abstract:

The abstract would benefit of a specific statement of the aim.

Introduction:

Also, make sure that the description of the PRIMARY AIM is consistent across abstract, introduction and methods, which is currently not the case. I also urge the authors as part of the aim to state the primary outcome and the timing of this.

Methods:

Please provide a reference for the following statement on page 7:

'The CBT-I will focus on the two components sleep restriction therapy and stimulus control,which have shown the highest efficacy for sleep improvement among components typically incorporated.'

The authors describe the intervention to be 'digital'. Please provide more information about the digital delivery of the intervention. Is this online, using an App on the phone or something else?

Statistics:

While I appreciate the authors effort to be very descriptive in the statistics section, I would suggest to be more direct and precise, as the many words actually are somehat confusing. For instance, I understand the authors want to use a linear mixed model to assess their primary outcome. Perhaps this could be described as:

'We will use a linear mixed model approach to compare the change in average NRS pain from baseline to 7 days post surgery between the CBTI and sleep education group. In the linear mixed model we will consider Treatment condition (CBTI and sleep education), time (baseline, 7 days and 6 mths.) and the interaction between Treatment condition and time

(Treatment conditionXtime) as fixed effects, and participants as random effects'. In case this is what the authors intend to do.

Also, it is not clear to me what the primary end point time is. It seems to be several, however this is not mirrored in the sample size estimation as no adjustment has been done for multiple outcomes/time points.

I think the Power calculation could also be more clear. How large a difference in change between groups is the study powered to detect for the primary outcome/endpint? This is not clear. Also, it is Uunclear if 100 is total smaple size or if this is what is needed in each group (i.e. total sample size=200)?

Discussion

Did the authors consider that many patients undergoing THA or TKA are opioid users? This does not seem to be an inclusion criteria, however this could interfere both with sleep and pain. I think could be considered/discussed.

In the study, the target is sleep but the primary outcome is pain. The rationale for this is briefly touched upon in the introduction, however I think this merit mentioning in the discussion. In case no effect is seen on pain, the critique will surely be that this was not the target of the intervention.

Congratulations on a great study idea and I wish you all the best with completing the study!

VERSION 1 - AUTHOR RESPONSE

In response to Reviewer (R) 1, Dr. Melanie R. Simpson, Norwegian University of Science and Technology

Bjurström and co-authors describe an interesting and well planned randomised control trial that seeks to assess the effect of cognitive behavioural therapy for insomnia (CBT-I) on postoperative pain control in the first days postoperatively and 6-months after total knee arthroplasty or total hip arthroplasty. The secondary outcomes include a large range of assessments ranging from changes in quantitative sensory testing variables (e.g., temporal summation, conditioned pain modulation), sleep, cognition (e.g., attention, memory, processing speed, executive function), mental health, health-related function, physical activity, quality of life, and blood biomarkers. The rationale and general design of the trial are clear and suitable. My main comments are regarding clarifications around the randomisation, sample size calculations and statistical analysis strategies.

R1.1: Randomisation: It is described that the randomisation procedure will ensure equal

proportions of participants undergoing THA and TKA. I have interpreted this to mean that the randomisation will be stratified by surgical procedure (later described as "Patient Group"). Is this correct? Will there be (or is there) block randomisation to ensure ongoing balance in numbers? This is not necessary, but the description implies a greater control of distribution of THA and TKA participants than a simple randomisation may end up giving, so it would be good to clarify.

A1.1: Yes, patients will be randomized within surgery type to generate equal distribution of conditions. To clarify, we have added the following text under “Randomization sequence generation” on P18: “The allocation sequence will be created via a computerized random number generator with equal probability to the interventions (1:1) within blocks by surgery type.”

R1.2: It is unclear how many sites are included in the study. Under "Study setting" it is described that surgeries will be performed at the Elisabeth hospital Uppsala or Enköping hospital, but also that "All three involved hospitals are in urban settings". Is the third hospital the Pain Center at Uppsala University Hospital where patients from both of the the surgery-performing hospital will be followed up? If there are multiple sites, will / is the randomisation stratified by site.

A1.2: Thank you for identifying this point which needs clarification. At this stage, we are collaborating with two sites where surgeries are performed, as stated under Study setting on P6. You are correct, the third hospital is the Pain Center at Uppsala University Hospital where we carry out the three study visits and procedures. The “urban settings” sentence was added to comply with SPIRIT guidelines. As we already write under Recruitment and retention on P18, “If recruitment proves to be complicated, efforts can be made to expand inclusion to additional nearby elective orthopedic surgical centers”. Hence, we might end up working with more than the two current centers. To clarify, we have edited the text under Study setting on P6: “On-site study visits will take place at the Multidisciplinary Pain Center at Uppsala University Hospital (Uppsala, Sweden). Surgeries will be performed at the Elisabeth hospital (Uppsala, Sweden), and the hospital in Enköping (Sweden), which are both located in urban settings.” Randomization will not be stratified by site, but we plan to conduct sensitivity analyses according to site.

R1.3: Secondary outcomes: there are many secondary outcomes described, and many of them have subscales, or can be analysed both as continuous scales or with previously

defined cuf-offs. If the research already considers some of these outcomes to be of greater interest / importance than others, I recommend more clearly defining the key secondary outcomes and what are considered more exploratory.

A1.3: Thank you for highlighting this issue. Indeed, there are many secondary outcomes in our study. Based on your comment, we have edited Table 2 (Outcome measures), and added a new subsection “Exploratory outcome measure” under which we list five of the outcomes which were previously termed secondary outcomes. The secondary outcomes are listed in a hierarchical order, to signal which are “key”, of most interest, but we would prefer to avoid further subdivision of these outcomes. Moreover, we will update the clinicaltrials.gov protocol to ensure that outcome measures are identical between the manuscript and the trial registry. Due to power considerations we will mostly be using continuous scales in our analyses.

R1.4: General comment on statistical methods: With two primary outcome points, many secondary outcomes, a number of choices required in the analysis strategy, it is advisable to write a "Statistical Analysis Plan" prior to analysis and to be finalised before unblinding of the dataset. This is a requirement for some journals. See: https://www.equator-network.org/reporting-guidelines/guidelines-for-the-content-of-statistical-analysis-plans-in-clinical-trials/

A1.4: Thank you for this suggestion and the link to the checklist. This process will indeed strengthen the future quality of the results.

R1.5: Statistical methods, second paragraph: The following description is unclear - "The basic design is a 2 Treatment Condition (CBT-I vs education control) by 2 Patient Group (TKA and THA), independent balanced analysis of variance (ANOVA) with one or more repeated outcome measures (Time) analyzed with linear mixed models (LMM)." Firstly, the 2 x 2 design is usually used to describe a trial with two randomly assigned treatment conditions. Thankfully you are not randomising surgical procedure, so the description of a two-arm, parallel-group design which is found in the Abstract seems more correct. As stated the main effect of interest is that of treatment condition, with a secondary plan to investigate the modifying effect of surgical procedure on the effect of treatment. The second unclear aspect of the sentence above is that it implies that ANOVA is a part of the design of the trial, whereas both ANOVA (with repeated measures) and LMM are both analysis strategies. Based on the rest of the paragraph, it becomes clear that LMM is the planned analysis

strategy, but remains unclear whether the use of ANOVA is also planned.

A1.5: We apologize for the confusion created here. Indeed, as the reviewer surmised, the statistical plan will only utilize mixed models ANOVA. This has been clarified in the "Statistical methods" section on P19-20. For example, on P19: "The basic design is a parallel group (Patient Group: TKA and THA), two arm (Treatment Condition: CBT-I vs education control), independent balanced analysis of variance (ANOVA) with one or more repeated outcome measures (Time) analyzed with linear mixed models (LMM). The number of repeated measures varies by assessment; for the primary analysis of NRS scores, the individual scores will be modeled rather than an average."

R1.6: For the primary analyses, it would be interesting to have a more detailed description of the LMM model - For example, will daily pain scores be included, or an average across each of the two 7-day periods? Will the model include a main effect of treatment, or estimate a common baseline and only post-randomisation differences between treatment groups (as recommended in Twisk et al 2018: https://www.sciencedirect.com/science/article/pii/S2451865417301849)? Surgery type will be included as a covariate, will other covariates be considered?

A1.6: Daily pain scores will be included in the model rather than averaged. We appreciate the suggested reference. Our original intention was a longitudinal analysis but the model without the treatment factor does seem appropriate.

Any other covariates such as would only be included in exploratory sensitivity analyses.

R1.7. Sample size calculation / power calculations (last paragraph in Statistical methods): Generally, this paragraph could be described more clearly:

a) It is unclear what is meant by "The lower limits of statistical power are focused within the primary analyses". Does this mean that the primary outcomes were used to calculate required sample size based on a minimum desired statistical power?

A1.7a: Correct. We believe this is now better described in "Statistical methods" on P20.

b) The [primary] outcomes described in this paragraph are sleep and pain at visit 2, post-op day 1-7 and 7-day periods at 3, 6 and 12 months. The methods section otherwise implies

that only pain on day 1-7 and at 6 months are primary outcomes. Were both sleep and pain and all follow-up time points considered in sample size calculations, or only some of these?

A1.7b: We have decided to consider the analysis of all long term follow up periods as secondary. Also, the text has been revised to clearly show that sleep measures are secondary outcomes. The sentence on P20 now reads: “Specifically, the key outcomes are the changes from baseline (Visit 1) in the pain measures to post-treatment (Visit 2) and immediate post-surgery (POD1-7).” While the modeling considered various combinations of immediate and durable effects, the literature only provided estimates of immediate effects. The power calculations remain appropriate for only assessing immediate effects of the treatment.

c) Was any adjustment made for the use of two primary outcomes (pain at day 1-7 and pain recorded over 7-days at 6 months)

A1.7c: No adjustments were made per se as we did model different effect patterns over time simultaneously. We have however reconsidered the expectation of any long-term effects; given that these have yet to be assessed in the literature that formed the basis for the power calculations, we will now test them as secondary outcomes.

d) Simulations seem like a very good strategy with this complex dataset, but it is unclear what level of pain reduction was considered to be clinically important in the calculations / what was the effect size the calculations were based on. Was the effect size in the sample size calculations defined based on findings from previous studies, and were these findings on what is clinically important or what CBT-I has previously achieved of difference in pain-scores? If the sample size is based on previously achieved differences in pain scores after CBT-I, are these clinically significant differences? If the sample size is based on MSD, can these be related to expected differences based on baseline pain scores and standard deviations?

A1.7d: The power calculations were based upon previously observed differences, which tend to be smaller than the generally agreed upon clinically meaningful differences for the pain NRS which is a change of approximately 2 points or more. There would certainly be sufficient power to observe such a large effect and, unlike the literature which has focused on chronic pain conditions, our expectation is that a MCID reduction of postoperative pain intensity might be achieved. While this approach might argue that a reduction in sample size could be considered, this would reduce power for secondary analyses that more likely

benefit from larger sample sizes. Relevant references have been added to the section on P20 which describes power calculations. Changes in the text are outlined below under A2.7.

e) Is the required sample size n=100 per group, or total?

A1.7e: The required sample is 100 total. As noted elsewhere, we do not expect to have sufficient power within Patient Group and have dropped such analyses. We have clarified on P20, for example: “A total sample size n=100 (TKA, THA combined) was selected given the upper limit and some loss of follow up.”

f) It is realistic that only 12 of 100 participants are lost to follow-up?

A1.8f: We believe it is realistic, given that we don´t expect much loss to follow-up in the immediate perioperative setting, during which the primary outcome data are collected. Our experiences from the pilot trial support this notion. Moreover, according to our power calculation, samples of 63 to 88 are required for 80% power, and the upper limit, 88, was chosen to allow loss of follow-up (given n=100).

g) The following statement is unclear: "As the groups will be balanced by Patient Group, all main effects and interactions of this factor will all be tested with equal, maximized power for the study design." This also implies that the randomisation strategy will achieve balance (see comment on stratification and block randomisation above). I am unsure if including an interaction term between treatment group and surgical procedure (i.e. Patient Group) will provide greater statistical power in the estimation of the main effect of treatment. I would be interested in a reference for this statement.

A1.8g: We have revised this section since, within this particular trial, we would not expect a strong interaction rather than strong main effects. In fact, it would be optimal in terms of generalization if there were near zero interactive effects. As there is no clear expectation of effect sizes for the Patient Group factor or the interaction of same with Treatment condition, we have simply confirmed sample size for the study was determined with the primary outcome for the Treatment Condition main effects over time in mind.

h) Will the described "Within Patient Group analyses" aim to estimate the effect of surgical procedure on pain level, or the modifying effect of surgical procedure on the effect of treatment on pain level.

A1.8h: We will no longer implement within Patient Group analyses for this trial. The statistical power is likely to be inadequate however, obviously if large unexpected main effects of

Patient Group or interactions with Treatment Condition are observed, the analysis by design will certainly detect them.

In response to Reviewer 2, Dr. Jonas Thorlund, University of Southern Denmark

Thank you for the opportunity to review this well-written and detailed protocol for a trial comparing CBTI with sleep education for postoperative pain in patients undergoing THA or TKA for osteoarthritis. I think this is a very relevant study,
Given the study is already ongoing my comments are mainly related to improving the description of the study in the protocol.

A: Thank you for these warm words about our study, we greatly appreciate it! We just want to clarify that a pilot version of the study, evaluating the CBT-I treatment, study flow and procedures, is ongoing, whereas the randomized, controlled trial has not yet started.

R2.1: Abstract:
The abstract would benefit of a specific statement of the aim.

A2.1: We completely agree and have added the following sentence to the Abstract: "The main objective is to investigate whether preoperative insomnia treatment in patients scheduled to undergo total knee arthroplasty (TKA) or total hip arthroplasty (THA) may improve acute postoperative pain control."

R2.2: Introduction:
Also, make sure that the description of the PRIMARY AIM is consistent across abstract, introduction and methods, which is currently not the case. I also urge the authors as part of the aim to state the primary outcome and the timing of this.

A2.2: Thank you for identifying this issue. We have removed "improves preoperative sleep" from the description of main objectives under Methods and analysis – Study overview on P6 and rephrased the sentence, which now reads: "The main objective is to evaluate whether preoperative insomnia treatment in patients with severe knee or hip osteoarthritis and comorbid insomnia, scheduled to undergo primary TKA or THA, improves acute postoperative pain control." The primary objective should now be consistently reported across Abstract (now added, see A2.1) – Introduction (P5: "...the main objective of the

PROSAP-A trial is to investigate whether preoperative sleep-promotion may lead to improved acute postoperative pain control.") – and Methods (see above).

The primary outcome is now clearly outlined in the Abstract: "The primary outcome measure is acute postoperative pain intensity, averaged over the first 7 postoperative days [POD]", as well as in the Methods section – Outcomes – Primary outcome on P9, and Table 2 which lists all outcomes.

R2.3: Methods:
Please provide a reference for the following statement on page 7:
'The CBT-I will focus on the two components sleep restriction therapy and stimulus control, which have shown the highest efficacy for sleep improvement among components typically incorporated.'

A2.3: We have added the following reference (new reference 32): Steinmetz L et al, Network meta-analysis examining efficacy of components of cognitive behavioural therapy for insomnia. Clinical Psychology Review 2024.

R2.4: The authors describe the intervention to be 'digital'. Please provide more information about the digital delivery of the intervention. Is this online, using an App on the phone or something else?

A2.4: The digital CBT-I intervention is described in the reference which we have provided (#33, Jernelöv et al). However, to clarify the "digital" aspect to the reader, we have amended the text under Cognitive behavioral therapy for insomnia on P7-8: "The treatment will consist of a 4-week self-guided digital, online (web app) CBT-I intervention (provided by the eHealth core facility at Karolinska Institutet, according to Jernelöv et al.)"

R2.5: Statistics:
While I appreciate the authors effort to be very descriptive in the statistics section, I would suggest to be more direct and precise, as the many words actually are somehat confusing. For instance, I understand the authors want to use a linear mixed model to assess their primary outcome. Perhaps this could be described as:
'We will use a linear mixed model approach to compare the change in average NRS pain from baseline to 7 days post surgery between the CBTI and sleep education group. In the linear mixed model we will consider Treatment condition (CBTI and sleep education), time (baseline, repeated 7 days) and the interaction between Treatment condition and time

(Treatment conditionXtime) as fixed effects, and participants as random effects'. In case this is what the authors intend to do.

A2.5: Thank you for this thoughtful comment. We have attempted to clarify above, see A1.5. The current way we phrase this on P19 is: "The basic design is a parallel group (Patient Group: TKA and THA), two arm (Treatment Condition: CBT-I vs education control), independent balanced analysis of variance (ANOVA) with one or more repeated outcome measures (Time) analyzed with linear mixed models (LMM). The number of repeated measures varies by assessment; for the primary analysis of NRS scores, the individual scores will be modeled rather than an average."

R2.6: Also, it is not clear to me what the primary end point time is. It seems to be several, however this is not mirrored in the sample size estimation as no adjustment has been done for multiple outcomes/time points.

A2.6: As noted above the power calculation is based upon the immediate postsurgical period and the ranking of outcomes has been better brought into alignment.

R2.7: I think the Power calculation could also be more clear. How large a difference in change between groups is the study powered to detect for the primary outcome/endpoint? This is not clear. Also, it is unclear if 100 is total sample size or if this is what is needed in each group (i.e. total sample size=200)?

A2.7: We have clarified and expanded the section about sample size calculations on P20: "The sample size calculations are focused within the primary analyses. Specifically, the key outcomes are the changes from baseline (Visit 1) in the pain measures to post-treatment (Visit 2) and immediate post-surgery (POD1-7). GLM simulations using data ranges based upon published studies[1 28 74] to estimate the effect sizes of the CBT-I treatment on insomnia (medium to large[28 75]) and the impact of the reduction of insomnia on measures of pain (medium[1 74]) suggest that samples of 63 to 88 are required for 80% power (α=.05, two-tailed); these simulations considered various combinations of effects that represented a range of small (d=.25) to medium (d=.55) effects over the eight key outcome assessments. A total sample size n=100 (TKA, THA combined) was selected given the upper limit and some loss of follow up. The estimated effect size is smaller than a two-point change in the NRS (0-10) which is commonly considered the minimal clinically important difference (MCID) for acute postoperative pain intensity after THA or TKA.[76] Secondary analyses examining the additional variables and timepoints will be identical in execution, however the impact of CBT-I may be reduced with lower statistical power."

R2.8: Discussion

Did the authors consider that many patients undergoing THA or TKA are opioid users? This does not seem to be an inclusion criteria, however this could interfere both with sleep and pain. I think could be considered/discussed.

A2.8: Preoperative opioid use is relatively rare in a Swedish context. While we agree that opioid treatment can clearly impact both sleep and pain, we believe implementing specific daily oral morphine equivalent levels as inclusion/exclusion criteria wouldn´t improve the study design. Instead, if we end up with a substantial subgroup of preoperative opioid users, we plan to conduct sensitivity analyses.

R2.9: In the study, the target is sleep but the primary outcome is pain. The rationale for this is briefly touched upon in the introduction, however I think this merits mentioning in the discussion. In case no effect is seen on pain, the critique will surely be that this was not the target of the intervention.

A2.9: This is a great point, and we have now added content in the Discussion highlighting that pain is an “off-target” outcome in the proposed study, but there is a strong theoretical and methodological basis to expect a positive outcome given the centrality of sleep to the defined postoperative recovery period.

Discussion, P23: “It is notable that pain is an “off-target” outcome of an intervention designed explicitly to improve sleep. Although ample experimental and longitudinal evidence now indicates that sleep disturbance prospectively predicts increased pain severity in chronic pain populations,[4, 78-79] trials of CBT-I in patients with chronic pain have resulted in weak and variable effect sizes on pain outcomes.[23, 80] Our study, however, will evaluate pain changes following CBT-I in a novel clinical context (recovery from knee surgery) that may foster unique effects. Given the centrality of sleep to surgical recovery and the well-defined post-operative recovery period, there is a strong theoretical and methodological basis for hypothesizing that sleep improvements following CBT-I will yield pain reductions in the present study. This contrasts with studies of CBT-I in chronic pain, in which sleep and pain changes are typically evaluated in samples composed of individuals who have experienced chronic pain in time arcs spanning months to decades.” References 78-80 have been added to the revised version.

VERSION 2 - REVIEW

Reviewer **2**

Name Thorlund, Jonas

Affiliation University of Southern Denmark, Institute of Sports Science and Clinical Biomechanics

Date 09-Jun-2025

COI

Thank you for responding to all my questions/comments. Good luck with the study.
